# Epicardial Unipolar Lead Loss of Ventricular Capture during Radiofrequency Ablation of Atrial Fibrillation

**DOI:** 10.1155/2020/4312315

**Published:** 2020-01-20

**Authors:** Daniel J. Chu, Wilson W. Lam

**Affiliations:** ^1^Cardiology Section, Department of Medicine, Baylor College of Medicine, Houston, TX 77030, USA; ^2^Texas Heart Institute, Baylor St. Luke's Medical Center, Houston. TX 77030, USA

## Abstract

*Introduction.* Abnormal pacemaker behavior can occur during radiofrequency ablation. The behaviors are varied and include loss of capture. The mechanisms in this context have not been well described in the literature. We describe a case of epicardial unipolar lead loss of ventricular capture during pulmonary vein isolation. *Case History.* A 48-year-old man with an epicardial dual chamber pacemaker and persistent atrial fibrillation presented for radiofrequency ablation (RFA) of his abnormal rhythm. During RFA, intermittent loss of ventricular capture was witnessed. Review of the device settings prior to and after the procedure showed an increase in ventricular threshold after the procedure. Loss of capture was shown to be dependent on location and RF energy delivered. It was independent of QTc and independent of local cellular changes that would increase threshold.

**Conclusion:**

We hypothesize the mechanism of loss of ventricular capture in this patient with an epicardial pacemaker with unipolar leads is related to intermittent shunt of voltage from the pulse generator to the grounding pad rather than the unipolar lead.

## 1. Introduction

Since the inception of the wearable pacemaker in 1957, cardiac implantable electronic devices (CIEDs) have become increasingly common in medicine [1]. In the modern era of electrophysiology, devices, along with radiofrequency ablation (RFA), comprise two of the main therapeutic options available for treating arrhythmias [2–4]. Invariably, more patients with permanent pacemakers are needing RFA. Despite this, there is a paucity of literature on the topic to base recommendations.

Early experience of the effect of RF energy on implanted pacemaker behavior demonstrated unusual responses in more than 50% of cases [5]. These responses ranged from oversensing, inappropriate pacing, reversion to noise mode, and loss of ventricular capture. Thought to be due in part to the impact of RF energy on the pulse generator, the mechanism of dysfunction remains incompletely understood.

To date, there is only a single case report as part of a series from Sadoul et al. [6] to describe transient loss of capture during RFA in a patient with a permanent pacemaker [5]. We report this case of epicardial unipolar lead loss of ventricular capture during RFA in order to add to the existing literature on this subject and contribute to the process of better understanding the causes of these abnormal device behaviors.

## 2. Case Presentation

A 48-year-old man with a history of congenital ventricular septal defect (VSD), complete heart block, and atrial fibrillation presented for pulmonary vein isolation. He underwent VSD repair as a child; however, the surgery was complicated by complete heart block. As a result, the patient had an epicardial dual chamber pacemaker. The initial epicardial dual chamber system was implanted in 1973; however, it was completely replaced in 1995. Thus, his current unipolar leads were in place since 1995. Since then, he had undergone several generator changes. His present system consisted of an atrial unipolar lead (Medtronic 4965), a ventricular unipolar lead (Medtronic 4965), and pulse generator (Medtronic Adapta ADDR01, DDD).

On presentation, his baseline rhythm was atrial fibrillation with ventricular pacing. Prior to the procedure, the device was reprogrammed to VOO 70 to avoid inhibition from interference with ablation. Ventricular threshold was confirmed to be 1.25 V @ 1.5 ms and output was set at 2.5 V @ 1.5 ms. Grounding pads were placed on the patient's back between the sacrum and the level of the umbilicus.

**Figure 1:**
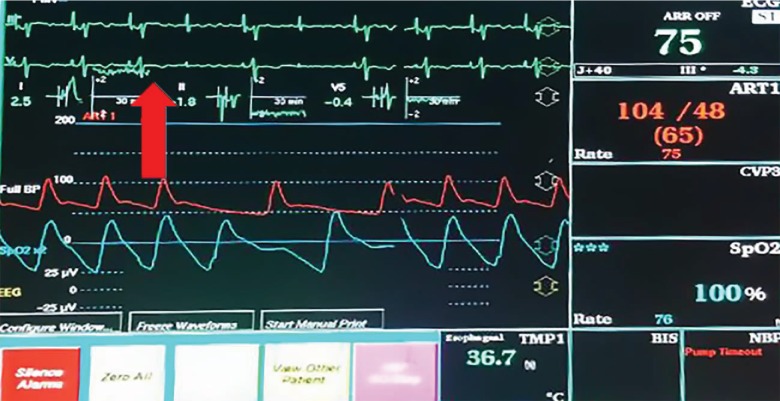
Monitor demonstrating intermittent loss of ventricular capture during RFA (arrow).

During wide antral circumferential ablation of the right upper pulmonary vein (RUPV), most distant to the ventricular epicardial lead, however closest to the grounding pad, intermittent loss of ventricular capture for single beats was noted (Figure 1). This was not present with ablation of the left-sided veins, roof, or right lower pulmonary vein. During ablation with 45 W at the RUPV, ventricular threshold increased to 3 V @ 1.5 ms, prompting intermittent loss of ventricular capture. Subsequent delivery of ablations at 40 W, 30 W, and 20 W also had loss of ventricular capture, while delivery of 15 W did not. Notably, once RF energy was turned off, the device functioned well and there was no loss of capture.

We then moved the coronary sinus (CS) decapolar catheter to the right ventricular apex. A threshold of 1.4 mA @ 1 ms was determined, and the pacemaker was set to VVI 40. Ablation at 45 W power did not lead to loss of capture. However, the VVI pacemaker had ventricular pacing throughout suggesting a decrease in sensed R wave amplitude, which we observed. Of note, QTc was unchanged between loss of capture beats at the RUPV, nonloss of capture beats at the left sided veins, and nonablation beats around the RUPV.

The patient underwent successful pulmonary vein isolation, and his final rhythm was A-sensed, V-paced. His postop course was unfortunately complicated by a small right MCA stroke resulting in mild residual deficits.

## 3. Discussion

In this case, we noticed abnormal pacemaker behavior during pulmonary vein isolation in a patient with atrial fibrillation. Given our observations, we hypothesize that loss of ventricular capture in this patient with an epicardial unipolar lead is (1) location dependent —loss of ventricular capture occurred at the most posterior structure, furthest from the RV epicardial pacing lead, however closest in proximity to the arc to posterior-inferior grounding pad; and (2) energy dependent—thresholds increased for 20 W and higher but not for 15 W. Our findings support one hypothesis that abnormal pacemaker behaviors are related to the strength of the RF current delivered [5–7]. Additionally, they clarify the relationship of ventricular loss of capture with distance, suggesting that proximity to the electrical arc may result in this behavior, at least in unipolar leads. This mechanism is less likely to be the case in bipolar leads; however, which are tougher to drain as the energy is between two close points.

By moving the CS catheter to the RV apex, the objective was to see if the pacing energy was being stolen from the cardiac device to the grounding pad and therefore not reaching the unipolar lead, resulting in loss of capture. The bipolar pacing lead maintained capture throughout. Thus, if loss of capture were due to a metabolic issue throughout the myocardium resulting in increased thresholds, then the RV pacing would have had increased threshold while ablating at the RUPV which it did not.

Regarding the QTc, if there were local changes affecting repolarization, QT prolongation should have been observed, and it was not. If there was an autonomic effect that invoked an increase in threshold of all cells, then RV pacing threshold would have risen like the unipolar did. Since the RV captured with bipolar pacing but the unipolar did not, we suspect that current was stolen by the grounding pad.

Thus, we also demonstrated that loss of capture was (3) independent of cellular changes to increase threshold—as the RV decapolar threshold was unaffected during ablation and (4) independent of QTc interval—as QTc was unchanged at the same location when there was no loss of capture and at different locations with ablation.

### 3.1. Limitations

Generalizability of findings in this case is limited by the unique patient. Unipolar leads have become increasingly rare in patients as most leads today are bipolar. Similarly, epicardial leads are less common. Thus, these findings may only apply to a small population of people that is probably decreasing.

Additionally, we were not able to actually prove that energy was being shunted from the pulse generator to the grounding pad; however, we had a strong suspicion this was the case. This is therefore only a hypothesis of the mechanism of action, and additional studies can be done in the future to demonstrate this objectively.

Of note, ventricular pacing inhibition has been reported in the literature during VT ablation, and this is therefore not a completely novel topic. In that setting, it is more likely to occur with an endocardial as opposed to epicardial lead given proximity and the possibility of contact with the ablation catheter. In the case of our patient, however, the event occurred during pulmonary vein isolation with an epicardial dual chamber system. Additionally, the device was not inhibited. It was still pacing, but it needed more energy to capture as we suspect energy was being stolen.

## 4. Conclusion

In conclusion, just as ablation can lead to alterations in impedance and sensed R wave amplitude, we hypothesize the mechanism of loss of capture in this case is related to intermittent shunt of voltage from the generator to the grounding pad rather than the unipolar lead.

Though this mechanism only applies to unipolar leads which are increasingly uncommon, we believe the case expands on the current understanding of unusual pacemaker behavior due to RF energy and is therefore important. Future work should focus on collecting prospective data on abnormal pacemaker responses during delivery of RF current. In doing so, we may further define the underlying mechanism of abnormal CIED responses. Better understanding of these behaviors may ultimately contribute to the development of improved recommendations for these patients during these procedures.
